# Optimizing Feature Selection and Machine Learning Algorithms for Early Detection of Prediabetes Risk: Comparative Study

**DOI:** 10.2196/70621

**Published:** 2025-07-31

**Authors:** Mahmoud B Almadhoun, MA Burhanuddin

**Affiliations:** 1 Fakulti Kecerdasan Buatan dan Keselamatan Siber, Universiti Teknikal Malaysia, Melaka, Durian Tunggal, 75450, Malaysia, 60 194807552

**Keywords:** prediabetes, machine learning, feature selection, prediction, extreme gradient boosting, support vector machine, k-nearest neighbors

## Abstract

**Background:**

Prediabetes is an intermediate stage between normal glucose metabolism and diabetes and is associated with increased risk of complications like cardiovascular disease and kidney failure.

**Objective:**

It is crucial to recognize individuals with prediabetes early in order to apply timely intervention strategies to decelerate or prohibit diabetes development. This study aims to compare the effectiveness of machine learning (ML) algorithms in predicting prediabetes and identifying its key clinical predictors.

**Methods:**

Multiple ML models are evaluated in this study, including random forest, extreme gradient boosting (XGBoost), support vector machine (SVM), and *k*-nearest neighbors (KNNs), on a dataset of 4743 individuals. For improved performance and interpretability, key clinical features were selected using LASSO (Least Absolute Shrinkage and Selection Operator) regression and principal component analysis (PCA)*.* To optimize model accuracy and reduce overfitting, we used hyperparameter tuning with RandomizedSearchCV for XGBoost and random forest, and GridSearchCV for SVM and KNN. SHAP (Shapley Additive Explanations) was used to assess model-agnostic feature importance. To resolve data imbalance, SMOTE (Synthetic Minority Oversampling Technique) was applied to ensure reliable classifications.

**Results:**

A cross-validated ROC-AUC (receiver operating characteristic area under the curve) score of 0.9117 highlighted the robustness of random forest in generalizing across datasets among the models tested. XGBoost followed closely, providing balanced accuracy in distinguishing between normal and prediabetic cases. While SVMs and KNNs performed adequately as baseline models, they exhibited limitations in sensitivity. The SHAP analysis indicated that BMI, age, high-density lipoprotein cholesterol, and low-density lipoprotein cholesterol emerged as the key predictors across models. The performance was significantly enhanced through hyperparameter tuning; for example, the ROC-AUC for SVM increased from 0.813 (default) to 0.863 (tuned). PCA kept 12 components while maintaining 95% of the variance in the dataset.

**Conclusions:**

It is demonstrated in this research that optimized ML models, especially random forest and XGBoost, are effective tools for assessing early prediabetes risk. Combining SHAP analysis with LASSO and PCA enhances transparency, supporting their integration in real-time clinical decision support systems. Future directions include validating these models in diverse clinical settings and integrating additional biomarkers to improve prediction accuracy, offering a promising avenue for early intervention and personalized treatment strategies in preventive health care.

## Introduction

A prediabetic state is characterized by elevated blood sugar levels, considered as an intermediate stage between normal glucose metabolism and type 2 diabetes [1]. In individuals with a high risk of diabetes, cardiovascular disease, and kidney complications, early diagnosis and intervention in prediabetes is important for delaying or preventing progression to diabetes [2]. In spite of lifestyle interventions, adherence remains one of the biggest challenges, which necessitates early and accurate detection.

While biochemical markers like fasting glucose and glycated hemoglobin are valuable, they may not capture the full spectrum of prediabetes risk factors, resulting in missed diagnoses and delayed interventions. To address this, a wide set of predictors, including clinical and genetic data, needs to be incorporated. This issue can be overcome by machine learning (ML), which can analyze complex relationships between a broad range of biomarkers [3]. By leveraging ML algorithms, this study aims to enhance the accuracy of prediabetes risk assessment and early detection.

A feature selection technique such as LASSO (Least Absolute Shrinkage and Selection Operator) regression and principal component analysis (PCA) further optimizes these models by focusing on the most apropos predictors, as a consequence improving both efficiency and interpretability [4,5]. Additionally, it reduces model complexity and boosts prediction accuracy by eliminating irrelevant or unnecessary data in ML. Models based on the most impactful clinical features, such as BMI, age, low-density lipoprotein cholesterol (LDL-C), and high-density lipoprotein cholesterol (HDL-C), can capture underlying patterns linked with prediabetes [6].

This paper assesses and compares the predictive power of various ML algorithms such as random forest, extreme gradient boosting (XGBoost), support vector machine (SVM), and *k*-nearest neighbors (KNNs), inclusive of feature selection methods such as LASSO and PCA. We aim to identify the most effective model and feature selection technique for the detection of early prediabetes, ultimately contributing to highly accurate diagnostics and personalized prevention.

In this study, key predictors such as BMI, age, LDL-C, and HDL-C were identified, which may refine diagnostic criteria and help with targeted prevention. The findings emphasize the capability for ML-based tools to improve prediabetes management and foster better patient outcomes through early intervention.

Various ML models have been used in recent studies to enhance detection accuracy and identify key risk factors associated with prediabetes progression. These approaches underscore the potential of ML in developing effective and clinically applicable prediction models for prediabetes risk.

An important direction is using ensemble and decision tree–based models to predict prediabetes. A study by Liu et al [7] evaluated logistic regression, decision trees, random forests, and XGBoost to predict diabetes progression in older patients with prediabetes. XGBoost was the most accurate model (60.66%), but its generalizability was limited by the dataset’s narrow demographic scope. In spite of a minor decline in predictive performance over time, XGBoost showed promise as a model for identifying prediabetes risk factors among older adults. Similarly, Abbas et al [8] developed a model of prediabetes risk score for a Middle Eastern cohort based on random forest, gradient boosting, XGBoost, and deep learning. This model effectively screens risk across different groups of individuals by analyzing demographic and physiological factors, including age, blood pressure, BMI, waist size, and gender. Primary care settings could benefit from the study’s focus on noninvasive, easily measurable variables.

Additionally, tree-based models, logistic regression, and LASSO have been commonly used to refine prediabetes risk prediction. Hu et al [9] developed a personalized nomogram that predicted 5-year prediabetes risk among Chinese adults. Using stepwise selection, LASSO, and ML models, Hu et al [9] found that the LASSO model provided the best performance with variables such as age, BMI, fasting blood glucose, and serum creatinine. As a result of this approach, LASSO can generate an accurate yet efficient model even with a limited number of predictive features. In another logistic regression–based study, Yu et al [10] validated a prediabetes assessment model on a large Chinese dataset. Based on C statistics and calibration plots, the model demonstrated good discrimination, but a cohort study might improve its performance.

Efforts have also been made to incorporate nonlaboratory risk factors into predictive models. In a study by Dong et al [11], lifestyle factors such as sleep duration and recreational activity were incorporated into a model using logistic regression and interpretable ML techniques, especially XGBoost. SHAP (Shapley Additive Explanations) was used to determine variable significance, revealing that lifestyle variables are crucial to the model’s detection efficiency. By incorporating clinical and lifestyle predictors, XGBoost can identify undiagnosed prediabetes and diabetes, offering a more comprehensive risk assessment.

As a result of these studies, we can observe that ensemble methods (random forest and XGBoost), regression-based approaches (logistic regression and LASSO), and interpretable ML models (eg, SHAP-enhanced XGBoost) all offer unique strengths in predicting prediabetes risk. According to the results, while tree-based models and ensemble models tend to be more accurate, regression techniques such as LASSO help create interpretable, efficient models, especially when resources are limited.

## Methods

### Dataset

This study used a dataset that is publicly accessible, which includes health records from 4743 individuals who were examined at the Health Management Center of Peking University Shenzhen Hospital from January 2020 to March 2023. The World Health Organization standards were followed when assessing fasting blood glucose levels, random blood glucose levels, oral glucose tolerance tests, and glycated hemoglobins of participants. Prediabetes was diagnosed if fasting blood glucose was between 6.1 and 6.9 mmol/L or if the blood glucose level was between 7.8 and 11.0 mmol/L after oral glucose tolerance test. Based on glucose metabolism status, participants were classified into 2 groups: normal (1593/4743, 33.6%) and prediabetes (3150/4743, 66.4%). The dataset included 22 features, comprising demographic, clinical, and laboratory variables such as age, BMI, HDL-C, and fasting blood glucose levels. The target variable for the study was binary, with participants categorized as either normal or prediabetic. Since this dataset is open to the public and anonymized, numeric values for individual IDs were preserved for traceability in the preprocessing phase, but they do not contain any personally identifiable information.

### Variable Assignment and Data Categorization

In this study, the dataset includes both categorical and numerical variables. The categorical variables, such as status, gender, urine glucose, and urine protein, were assigned specific values to facilitate analysis. These values allow for easy differentiation between groups or conditions. On the other hand, continuous or numerical variables, such as age, BMI, and various blood and urine biomarkers, were used as-is without specific value assignments since they naturally provide a range of measurements. Table 1 shows the assigned values for each of the categorical variables.

**Table 1.** Dataset variables and descriptions for prediabetes risk assessment.

Variable name	Meaning of variable	Type of variable	Assignment description
Status	Glucose metabolic status	Categorical variable	1=normal, 2=prediabetes
Age	Age	Numerical variable	Is unassigned
Gender	Gender	Categorical variable	0=female, 1=male
BMI	Body mass index	Numerical variable	Is unassigned
SBP	Systolic blood pressure	Numerical variable	Is unassigned
U-GLU	Urine glucose	Categorical variable	0=negative, 1=positive
PRO	Urine protein	Categorical variable	0=negative, 1=positive
TP	Total protein	Numerical variable	Is unassigned
ALB	Albumin	Numerical variable	Is unassigned
GLB	Globulin	Numerical variable	Is unassigned
T-BIL	Total bilirubin	Numerical variable	Is unassigned
DB	Direct bilirubin	Numerical variable	Is unassigned
IB	Indirect bilirubin	Numerical variable	Is unassigned
ALT	Alanine aminotransferase	Numerical variable	Is unassigned
AST	Aspartate transaminase	Numerical variable	Is unassigned
BUN	Blood urea nitrogen	Numerical variable	Is unassigned
SCr	Serum creatinine	Numerical variable	Is unassigned
UA	Uric acid	Numerical variable	Is unassigned
TC	Total cholesterol	Numerical variable	Is unassigned
TG	Triglycerides	Numerical variable	Is unassigned
HDL-C	High-density lipoprotein cholesterol	Numerical variable	Is unassigned
LDL-C	Low-density lipoprotein cholesterol	Numerical variable	Is unassigned

### Data Preprocessing

#### Overview

For improved model performance, data preprocessing involved handling missing values through mean imputation, balancing the dataset using SMOTE (Synthetic Minority Oversampling Technique), and scaling features with StandardScaler() and MinMaxScaler(). Through these steps, the dataset was optimized for building reliable ML models for prediabetes risk prediction.

#### Handling Missing Data

Missing values were imputed using the mean of the corresponding feature, guaranteeing consistency and completeness in the dataset.

#### Balancing the Dataset

The dataset has an imbalanced class distribution, with 33.6% (1593/4743) representing the normal group (status=1) and 66.4% (3150/4743) representing the prediabetes group (status=2). This type of imbalance can influence the performance of classification models, specifically incorrectly predicting the minority class (normal group in this case), so SMOTE was used to oversample the minority class (normal group). This step ensured that the ML models were not biased toward the larger class, improving predictive performance [12], particularly for prediabetes detection.

#### Scaling and Normalization

Scaling and normalization are pivotal steps when preparing continuous variables for models such as KNN, SVM, and LASSO, which are sensitive to feature scaling. To address this, the features are standardized using the “StandardScaler(),” which tunes them to have a mean of 0 and an SD of 1. This standardization guarantees that all features are on a similar scale and refines model performance. In addition, normalization can be applied using the “MinMaxScaler(),” which transforms the data into a range between 0 and 1 [13].

### Exploratory Data Analysis

**Figure 1.**
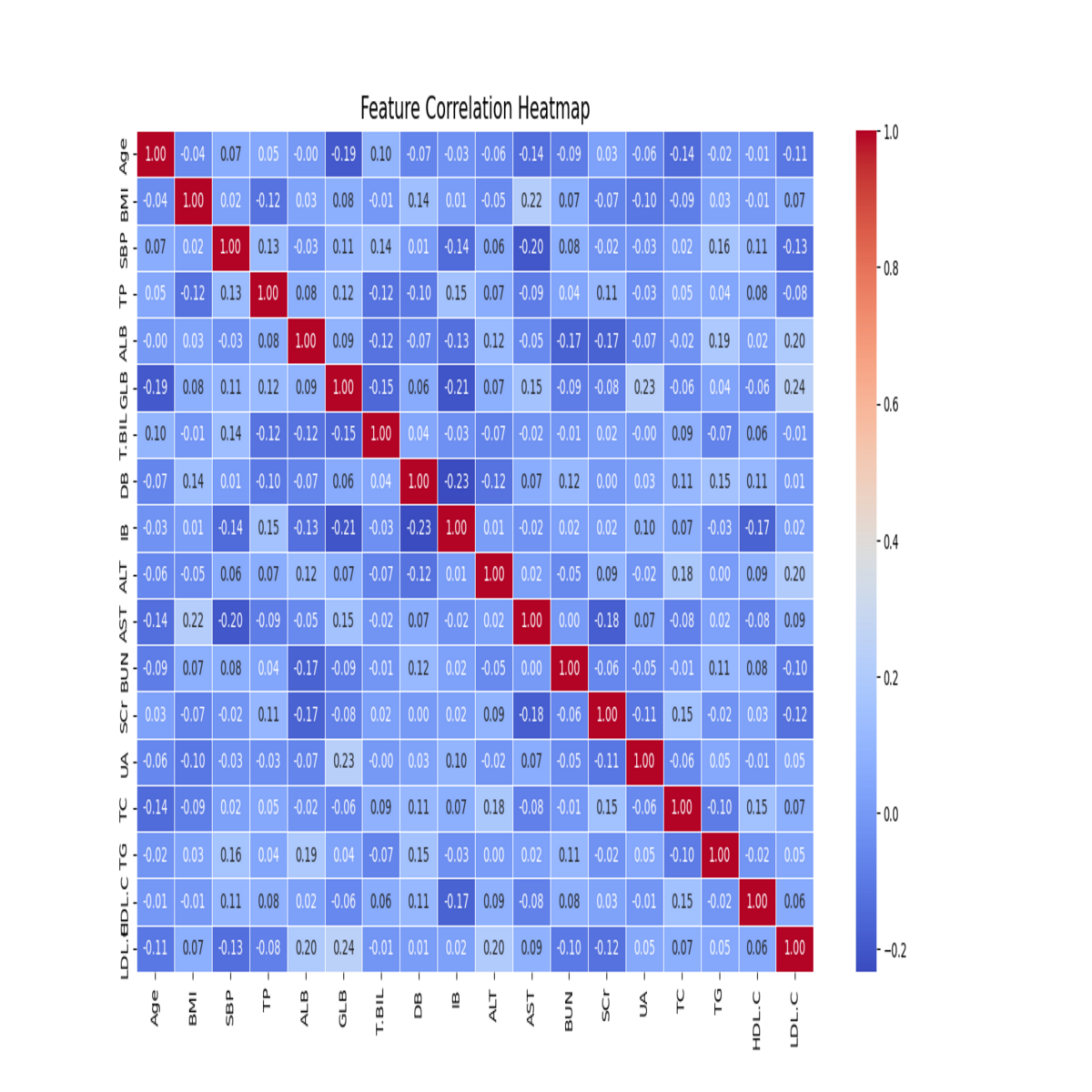
Heatmap distribution of the dataset features. ALB: albumin; ALT: alanine aminotransferase; AST: aspartate transaminase; BUN: blood urea nitrogen; DB: direct bilirubin; GLB: globulin; HDL-C: high-density lipoprotein cholesterol; IB: indirect bilirubin; LDL-C: low-density lipoprotein cholesterol; SBP: systolic blood pressure; SCr: serum creatinine; T-BIL: total bilirubin; TC: total cholesterol; TG: triglyceride; TP: total protein; UA: uric acid.

To obtain an understanding of the relationship across several features and to pick out any patterns, trends, or correlations that may guide next steps, the dataset was completely explored before applying predictive models. Heatmaps were used to visualize the relationship between numerical variables as shown in Figure 1. The main goal of this step is to gain a fruitful understanding of the raw data and arrange it for additional analysis [14]. Among the assessed models, SHAP analysis was performed solely on the XGBoost classifier due to its alignment with the TreeExplainer framework. Models based on trees benefit from SHAP’s precise additive feature attributions, which are computationally efficient and theoretically robust. XGBoost’s built-in support for SHAP made it more interpretable than other models (eg, SVM, KNN, and random forest).

### Features Selection

#### Overview

Two principal features selection techniques were applied after the data exploration phase to choose the most relevant and informative variables. A suitable feature selection not only enhances the performance and interpretability of a model but also reduces computational complexity and the risk of overfitting [15].

#### LASSO Regression

LASSO regression was used as the first method for feature selection. The LASSO method reduces the number of variables by shrinking the coefficients of less important features to zero, which effectively eliminates them from the model [16]. It is mostly useful for handling multicollinearity as it automatically picks one feature from a set of highly correlated features such as LDL-C and total cholesterol based on the correlation heatmap.

#### About PCA

The main aim of this technique is to reduce dimensionality in the dataset by transforming the base features into a smaller set of uncorrelated components while keeping most of the variance in the data [17]. In models facing overfitting, such as SVM and XGBoost, PCA reduced multicollinearity and compressed the retaining 95% of the variance in the data. Additionally, PCA reduced the number of variables, simplifying the model and making it more computationally efficient [18].

Before training the predictive models, these features selection techniques were applied. Using only relevant predictors improved model performance and generalizability. By using a structured approach to data exploration and features selection, we lay a strong foundation for building and evaluating ML models for prediabetes risk prediction in the next phase.

### Model Development

#### Overview

In this study, different ML models were used to predict the onset of prediabetes. These algorithms were selected due to their ability to handle high-dimensional data, interpretability, and performance in classification tasks. As well as each model was tuned and evaluated to optimize performance for prediabetes detection.

#### XGBoost

XGBoost is a powerful gradient-boosting algorithm that constructs an ensemble of decision trees to improve classification accuracy. Each one tree is sequentially trained to emend the errors of the previous trees, which makes it more powerful for tasks with complex relationships between features [19]. XGBoost is known for its performance and speed in handling big datasets, which makes it appropriate for medical prediction tasks like prediabetes diagnosis. In addition, XGBoost applies regularized boosting techniques to overcome the difficulty of the model and correct overfitting; as a result, increasing model accuracy [20].

#### Random Forest

Random forest is an ensemble learning approach that constructs numerous decision trees during training. Every tree is built using a random subset of features and data samples, and the last prediction is made by averaging the predictions from all trees [21]. Random forest minimizes the risk of overfitting by using a bagging approach and tends to accomplish well on classification issues such as prediabetes detection.

#### About SVM

SVM is a supervised learning model that separates data points into distinct classes by finding an optimal hyperplane. For the complex relationships between predictors, such as BMI and age, a nonlinear kernel was applied. This method is suited for medical diagnosis since the decision boundary is not linearly separable in high-dimensional spaces [22].

#### About KNNs

KNN is an uncomplicated, nonparametric classifier that specifies the class label based on the most votes of the KNNs in the feature space [23]. In this study, KNN was used after scaling the features, and the optimal number of neighbors was set through hyperparameter tuning. Despite KNN being computationally intensive for big datasets, its clarity and interpretability make it a beneficial model for prediabetes classification.

### Hyperparameter Tuning and Cross-Validation

#### Overview

Hyperparameter tuning was used for all models to recognize the optimal settings for each algorithm. To achieve that, we used GridSearchCV and RandomizedSearchCV, which systematically explore a range of hyperparameters and choose the set that maximizes model performance.

#### GridSearchCV

All combinations of hyperparameters are assessed exhaustively through a particular parameter grid. It is a systematic approach to identifying the effective parameter set [17]. With large datasets and complex models, it can be computationally expensive, so this study used GridSearchCV for models with a relatively small hyperparameter search space, which made it feasible to explore all combinations. The KNN algorithm was tuned by tuning the number of neighbors (*k*) and the distance metric.

#### RandomizedSearchCV

A randomized search of the hyperparameter space selects hyperparameter settings from the specified ranges [24]. It is more efficient than GridSearchCV when the search space is large because it explores a representative sample of possible combinations instead of testing them all. We used this technique for more complicated models such as random forest and XGBoost when the number of hyperparameters and possible values was too large for a wide search. RandomizedSearchCV assists with identifying optimal hyperparameters by setting a limit on the number of iterations (eg, 40).

### Tuning Process for Each Model

#### XGBoost

The hyperparameters, such as the maximum tree depth, the learning rate, and the subsample ratio, were tuned using RandomizedSearchCV. This approach allowed for a more efficient search through a vast range of parameter values, making it fit for models with big parameter spaces. Random sampling allowed the tuning process to explore a diversity of hyperparameter combinations while preventing overfitting and maximizing classification accuracy.

#### Random Forest

To optimize hyperparameters such as the number of trees, maximum tree depth, and minimum samples required for a part, RandomizedSearchCV was used. This approach is selected for random forest because of the large search space, as it can easily sample a subset of hyperparameters to explore near-optimal settings.

#### About SVM

To fine-tune hyperparameters such as the kernel type and penalty parameter C, GridSearchCV was used. Due to the smaller search space for SVM, GridSearchCV is considered the best choice because this approach performs a wide search over the specified parameter values, so it guarantees to find the best possible combinations for the model.

#### About KNNs

To tune the distance metrics (eg, Euclidean or Manhattan distance) and number of neighbors (*k*), the GridSearchCV method was applied. This approach is useful to pick out the most effective neighborhood size and similarity measures for predicting prediabetes.

This tuning strategy guaranteed that every model was fine-tuned to work optimally for prediabetes prediction.

### Cross-Validation Approach

The tuning process for each model included *k*-fold cross-validation to ensure reliable performance estimation and reduce the risk of overfitting. In *k*-fold cross-validation:

- The dataset is divided into *k* equal-sized subsets (folds).
- The model is trained on *k* – 1 folds and tested on the remaining fold. This process is repeated *k* times, with each fold serving as the test set once. The results are averaged to get a final evaluation metric.
- 5-fold cross-validation was used in this study, which balances computational cost and model evaluation reliability.

Through cross-validation, a robust estimate of model performance across various subsets of data is obtained by evaluating how well the model generalizes to unseen data [25]. To choose the best-performing parameter set, this method was used during hyperparameter tuning.

### Model Evaluation Metrics

#### Overview

To evaluate the performance of ML models, various metrics were applied.

#### Accuracy

This is the measure of the percentage of true predictions made by the model out of all predictions. Nevertheless, accuracy alone can be misleading, particularly when the classes are imbalanced, as in the case of prediabetes diagnosis.

#### Precision

The proportion of true positive predictions to the total number of positive predictions. High precision indicates that the model produces few false positive errors, which is important in minimizing irrelevant treatments.

#### Recall (Sensitivity)

The ratio of correct positive predictions to the total actual positives. A higher recall means fewer cases of prediabetes were missed, making it necessary for early prediabetes diagnosis.

#### *F*_1_-Score

The harmonic means of precision and recall contribute a balance between both metrics. It is mainly valuable when false positives and false negatives have serious consequences.

#### ROC-AUC Score

The ROC-AUC (receiver operating characteristic area under the curve) assesses the capability of the model to distinguish between both classes (normal and prediabetes). The ROC-AUC score provides an aggregate measure of performance throughout all classification thresholds, where a higher value refers to superior model performance.

#### Cross-Validated ROC-AUC

In addition to evaluating ROC-AUC on the test set, cross-validated ROC-AUC provides a more reliable estimate of the model’s ability to generalize. This metric was calculated using *k*-fold cross-validation, giving a better indication of how the model will perform on unseen data.

By using these evaluation metrics, the comparative performance of the ML models was assessed, with a particular focus on balancing accuracy, precision, recall, and *F*_1_-score to ensure reliable predictions for prediabetes risk assessment.

## Results

### XGBoost, Random Forest, SVM, and KNN

This section provides a comparative evaluation of the ML models applied in this study—XGBoost, random forest, SVM, and KNN—along with the results of feature selection techniques, such as LASSO regression and PCA. The performance of each model is assessed using multiple evaluation metrics, including accuracy, precision, recall, *F*_1_-score, and ROC-AUC scores, on both the test set and cross-validation. Table 2 shows the performance metrics comparison of the ML models.

**Table 2.** Performance metrics comparison of machine learning models.

Model	Accuracy (%)	Precision	Recall	*F*_1_-score	ROC-AUC^a^ (test set)	Cross-validated ROC-AUC
XGBoost^b^	74.7	0.8128	0.7889	0.8007	0.7930	0.8600
Random forest	75.9	0.8391	0.7169	0.7732	0.8030	0.9117
SVM^c^	73.9	0.6260	0.6686	0.6466	0.7791	0.8630
KNN^d^	70.8	0.6901	0.6881	0.6890	0.7845	0.8397

a ROC-AUC: receiver operating characteristic area under the curve.

b XGBoost: extreme gradient boosting.

c SVM: support vector machine.

d KNN: *k*-nearest neighbor.

### Model Performance Comparison

#### Overview

The following subsections present the comparative results of XGBoost, random forest, SVM, and KNN models, each fine-tuned using hyperparameter optimization and evaluated using key performance metrics.

#### XGBoost

Based on 5-fold cross-validation, the XGBoost model showed a cross-validated ROC-AUC score of 0.86, indicating powerful discrimination between normal and prediabetic cases. In addition, the model achieved a precision of 0.8128, a recall of 0.7889, and an *F*_1_-score of 0.8007 for the prediabetes class. This balanced performance emphasizes the model’s strength to effectively minimize both false positives and false negatives, making it an effective method of prediabetes detection.

#### Random Forest

The random forest model achieved an excellent performance with a cross-validated ROC-AUC score of 0.9117, demonstrating its capability to generalize well across various subsets of the data. The model demonstrated a precision of 0.8391, a recall of 0.7169, and an *F*_1_-score of 0.7732 for the prediabetes class. This indicates that the random forest model not only lowers the likelihood of false positives but also keeps a powerful recall rate, guaranteeing that fewer cases of prediabetes are missed.

#### About SVM

An SVM model, evaluated through 5-fold cross-validation, achieved a cross-validated ROC-AUC score of 0.8630, indicating its ability to distinguish between normal and prediabetic cases with high accuracy. For the prediabetes class, the model achieved a precision of 0.6260, a recall of 0.6686, and an *F*_1_-score of 0.6466. Despite the SVM model providing a moderate balance between precision and recall, its recall score indicates potential for missing fewer prediabetic cases, making it a feasible choice for early-stage diagnosis.

#### About KNNs

The KNN model, evaluated using 5-fold cross-validation, demonstrated a cross-validated ROC-AUC score of 0.8397, reflecting its ability to differentiate between normal and prediabetic cases with moderate effectiveness. The model recorded a precision of 0.6901, a recall of 0.6881, and an *F*_1_-score of 0.6890 for the prediabetes class. Although KNN performed slightly lower in terms of accuracy and precision compared to other models, it still provides an interpretable solution for prediabetes.

### Performance Enhancement Through Hyperparameter Tuning

To optimize the performance of SVM and KNN, we used GridSearchCV for hyperparameter tuning. For more complex models such as XGBoost and random forest, RandomizedSearchCV was used to efficiently explore broader hyperparameter spaces.

Tables3  4 highlight the improvement in model performance after hyperparameter optimization. All 4 models—XGBoost, random forest, SVM, and KNN—showed notable gains in both ROC-AUC and *F*_1_-score metrics. For instance, XGBoost’s ROC-AUC improved from 0.782 to 0.860, and random forest’s from 0.807 to 0.9117. These results confirm the effectiveness of using GridSearchCV and RandomizedSearchCV in tailoring model parameters to the dataset, ultimately boosting classification accuracy and robustness. This step is particularly critical for clinical applications, where small improvements in sensitivity or specificity can have substantial impacts on patient outcomes.

The parallel processing option n_jobs = –1 was used to enable parallel processing. Each model required 3-8 minutes to be tuned on a standard multicore computer.

**Table 3.** Hyperparameter tuning summary for all models.

Model and hyperparameter		Range or values tested
SVM^a^		
	C	[0.1, 1, 10]
	Kernel	['linear’, 'rbf’]
	Gamma (rbf)	['scale’, 'auto’]
KNN^b^		
	n_neighbors	[3, 5, 7, 9, 11]
	Metric	['euclidean’, 'manhattan’]
XGBoost^c^		
	n_estimators	[50, 100, 200, 300]
	learning_rate	[0.01, 0.05, 0.1, 0.2]
	max_depth	[3, 5, 7, 9]
	Gamma	[0, 0.1, 0.3, 0.5]
	Subsample	[0.6, 0.8, 1.0]
	colsample_bytree	[0.6, 0.8, 1.0]
Random forest		
	n_estimators	[50, 100, 200]
	max_depth	[None, 3, 5]
	min_samples_split	[2, 5]
	min_samples_leaf	[1, 2]
	max_features	['sqrt’, 'log2']
	Bootstrap	[True]

a SVM: support vector machine.

b KNN: *k*-nearest neighbor.

c XGBoost: extreme gradient boosting.

**Table 4.** Effect of hyperparameter tuning on model performance.

Model and metric		Default	Tuned (GridSearchCV/RandomizedSearchCV)
XGBoost^a^			
	ROC-AUC^b^	0.782	0.860
	*F*_1_-score	0.731	0.801
Random forest			
	ROC-AUC	0.807	0.9117
	*F*_1_-score	0.742	0.773
SVM^c^			
	ROC-AUC	0.813	0.863
	*F*_1_-score	0.591	0.646
KNN^d^			
	ROC-AUC	0.805	0.839
	*F*_1_-score	0.652	0.689

a XGBoost: extreme gradient boosting.

b ROC-AUC: receiver operating characteristic area under the curve.

c SVM: support vector machine.

d KNN: *k*-nearest neighbor.

### Descriptive Patterns From Exploratory Data Analysis Findings

#### Overview

Figure 1 shows several important patterns that emerged. The following features are highly correlated.

#### Strong Positive Correlation

Total cholesterol and LDL-C exhibited a strong positive correlation. As a result, the model may be redundant due to those variables sharing similar information. One of these features could potentially be excluded in the feature selection phase if it has a high correlation. It was found that total protein and albumin exhibit a high correlation, suggesting that combining them may not provide more insight than using either separately.

#### Weak or No Correlations

Correlations between variables such as age, BMI, and uric acid were weak or negligible. This is a significant finding because these variables may provide unique independent information that makes model-building more effective.

#### Negative Correlation

A mild negative correlation was found between LDL-C and HDL-C, which is consistent with their known inverse roles in cardiovascular health. Age and HDL-C also exhibited a slight negative correlation, suggesting that lipid profiles might change with aging. Multicollinearity issues happen when highly correlated variables distort the model’s ability to differentiate between them due to this exploration in sights. It is crucial to recognize such relationships early in the process so that multicollinearity can be handled, and redundant features can be dropped in the next step, features selection.

**Figure 2.**
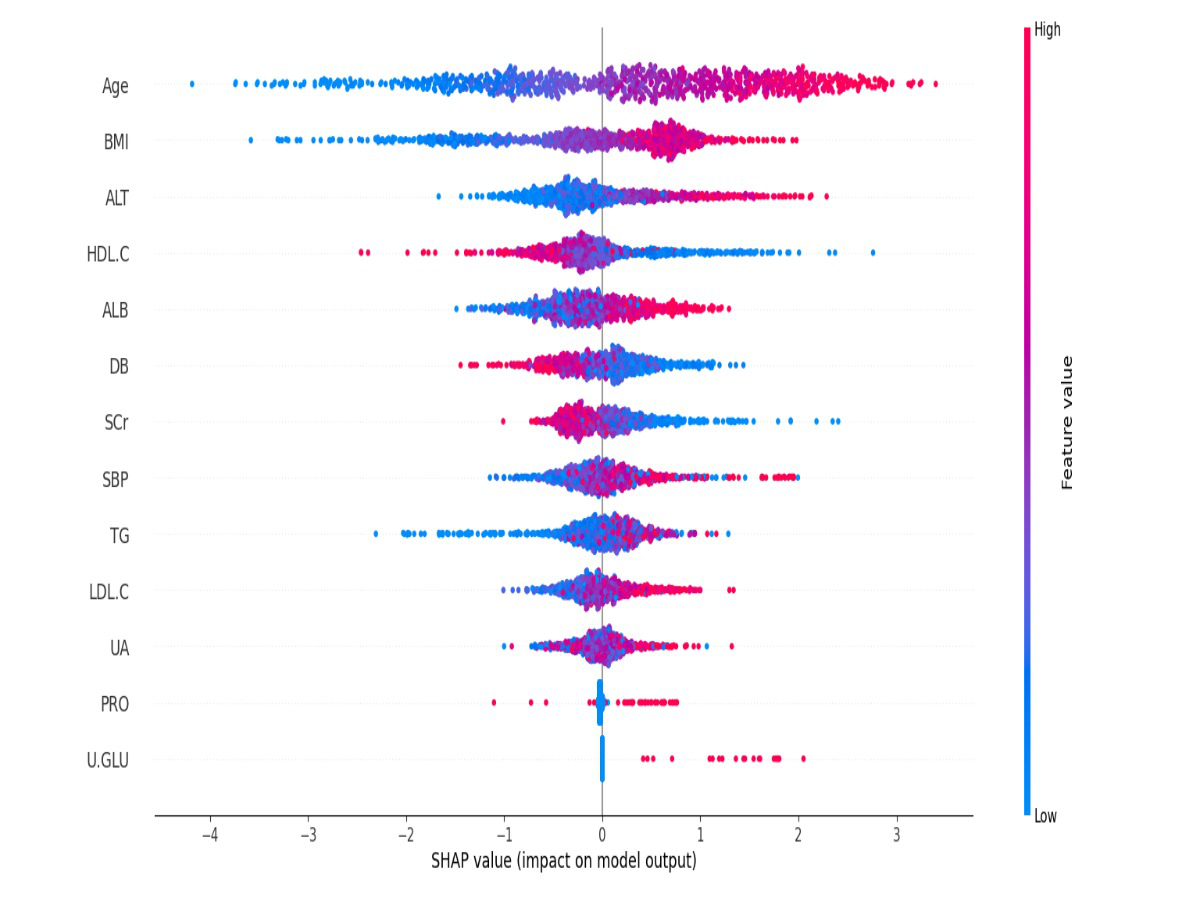
SHAP summary plot of XGBoost model. ALB: albumin; ALT: alanine aminotransferase; DB: direct bilirubin; HDL-C: high-density lipoprotein cholesterol; LDL-C: low-density lipoprotein cholesterol; PRO: urine protein; SBP: systolic blood pressure; SCr: serum creatinine; SHAP: Shapley Additive Explanations; TG: triglyceride; U-GLU: urine glucose; UA: uric acid; XGBoost: extreme gradient boosting.

A summary plot of SHAP data derived from the XGBoost model is shown in Figure 2. The most significant predictors are age, BMI, HDL-C, and LDL-C. As these variables are well-established risk factors for prediabetes, these findings support clinical intuition. Additionally, SHAP provided valuable visual confirmation that agreed with both the correlation analysis and the LASSO feature selection. Using these exploratory data analysis findings, LASSO regression and PCA were applied for feature selection, ensuring that informative predictors were retained while reducing redundancy and improving interpretability.



### Feature Importance and Selection

**Figure 3.**
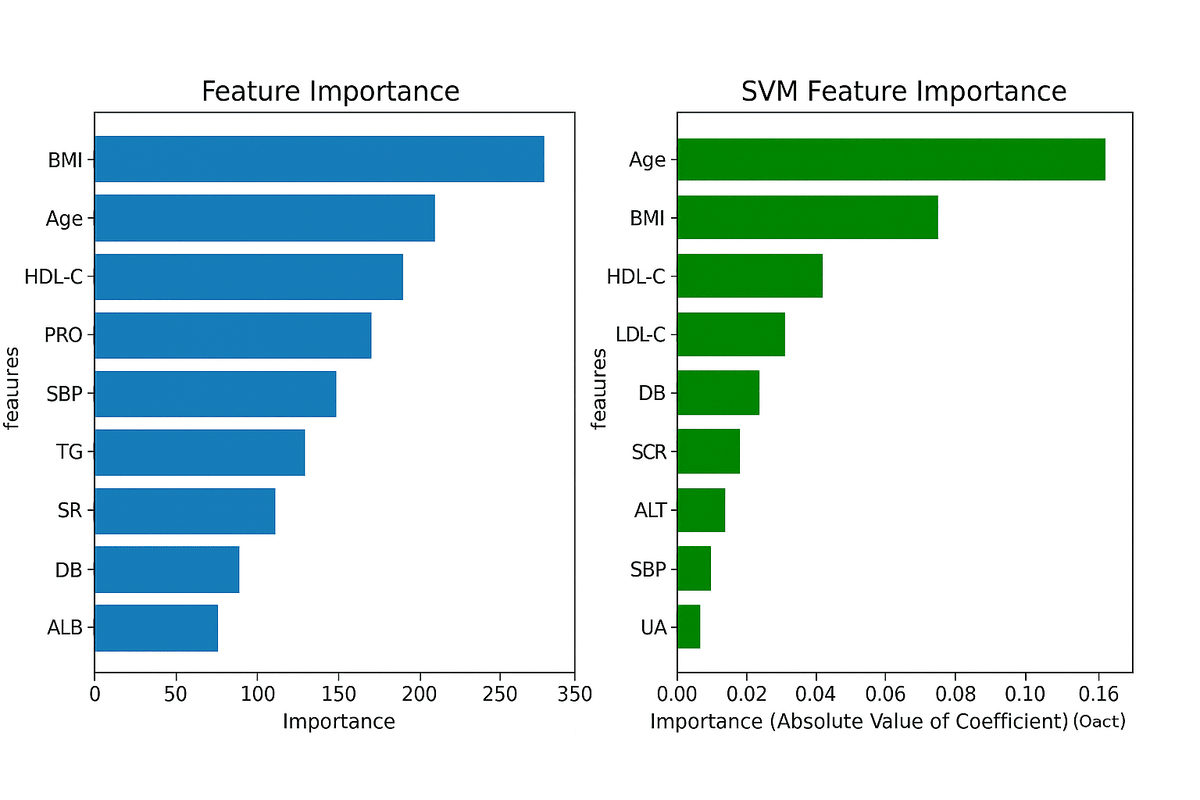
Features importance plots for XGBoost and SVM. ALB: albumin; ALT: alanine aminotransferase; DB: direct bilirubin; HDL-C: high-density lipoprotein cholesterol; LDL-C: low-density lipoprotein cholesterol; PRO: urine protein; SBP: systolic blood pressure; SCr: serum creatinine; SR: ; SVM: support vector machine; TG: triglyceride; UA: uric acid; XGBoost: extreme gradient boosting.

Feature selection over LASSO regression guaranteed that every model was trained on the most relevant predictors. During LASSO, features like BMI, age, and HDL-C were consistently identified as significant predictors of prediabetes as shown in Figure 3. These features were retrained in the final model because of their significant predictive power across different iterations. The models differed in which features they emphasized:

- XGBoost identified BMI as the most significant predictor, aligning with established research that links higher BMI with increased prediabetes risk.
- SVM prioritized age as the first predictor, indicating that age may play an additional critical role when nonlinear relationships between variables are considered.

- Random forest and KNN provide insights into other key features such as LDL-C and HDL-C, demonstrating the various aspects of the data that every algorithm emphasizes.

This variance in feature significance underscores the utility of designing diverse models and selection techniques to better understand the predictors of prediabetes risk.

### PCA Component Retention

PCA retained 12 principal components, accounting for 95% of the variance in the dataset.

### Confusion Matrices

#### Overview

**Figure 4.**
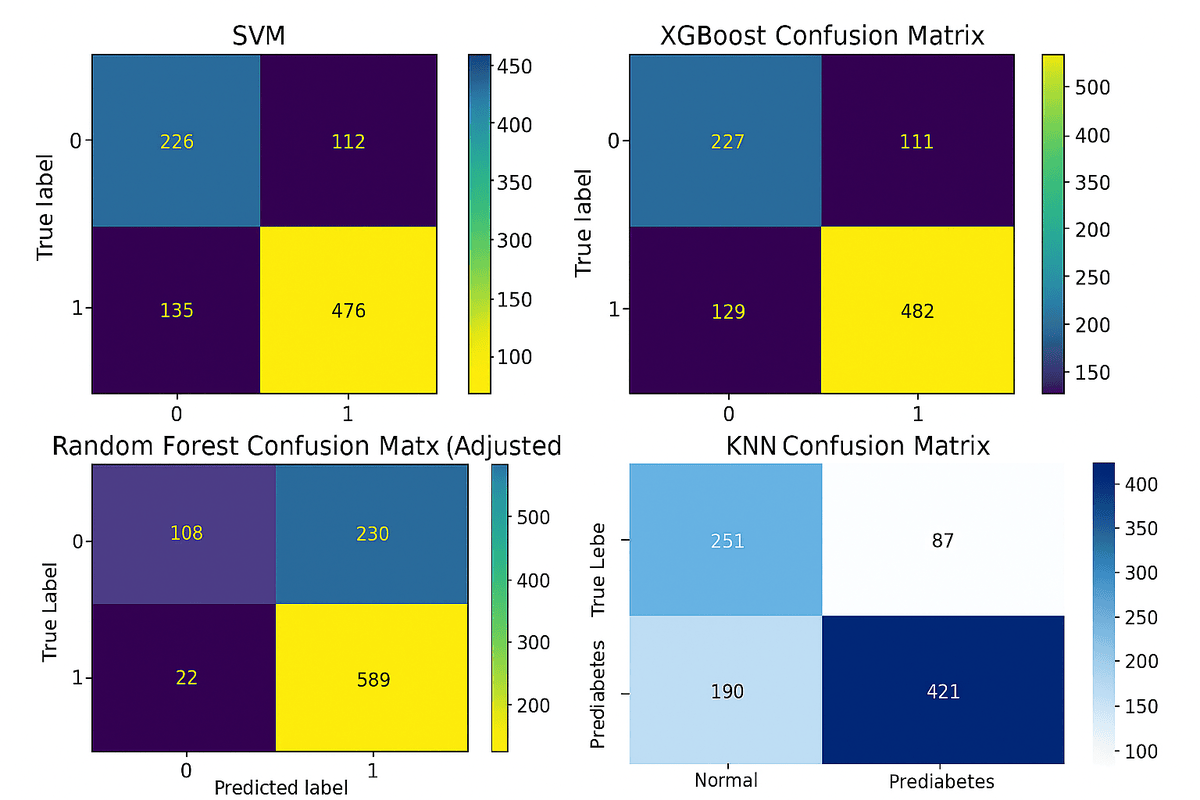
Confusion matrix for XGBoost, SVM, random forest, and KNN models. KNN: *k*-nearest neighbor; SVM: support vector machine; XGBoost: extreme gradient boosting.

As shown in Figure 4, the confusion matrix demonstrates that every model’s classification performance is detailed in terms of distinguishing normal cases from prediabetic cases. These results reflect the trade-offs each model faces in terms of true positives, false positives, true negatives, and false negatives.

#### XGBoost

A comparatively balanced classification was accomplished with the XGBoost model, with 482 true positives and 227 true negatives, referring to good sensitivity. However, it recorded 129 false negatives and 111 false positives, proposing some limitations in minimizing misclassification errors, especially false negatives, which are pivotal in clinical settings.

#### Random Forest

The random forest model (default threshold of 0.5) correctly identified 513 true positives and 208 true negatives, which are better results compared to XGBoost. The model demonstrated a higher sensitivity than other models, as it reduced the number of false negatives to 98. Despite this, 130 false positives were observed, which indicates a slightly higher trade-off in specificity.

A threshold adjustment of 0.2627 substantially improved the random forest’s ability to detect prediabetic cases, resulting in 589 true positives and 22 false negatives. A notable rise in false positives (230) and a reduction in true negatives (108) resulted from this adjustment, indicating a move toward maximizing sensitivity over specificity. There may be some advantages to this configuration in scenarios where minimizing missed prediabetic cases is prioritized over averting false positives.

#### About SVM

For the overall distribution of true positives and true negatives, the SVM model obtained 476 true positives and 226 true negatives, which is like XGBoost’s. A total of 135 false negatives and 112 false positives have been recorded, indicating that while SVM has a strong classification capability, it is more susceptible to false negatives, which limits its effectiveness for early detection cases.

#### About KNNs

This model performed moderately, generating 421 true positives and 251 true negatives. Even though KNN can effectively detect normal cases, it is less reliable when it comes to identifying prediabetic cases. It showed 190 false negatives and 87 false positives, indicating a higher rate of misclassification.

To summarize, the confusion matrices demonstrate that the random forest model minimizes false negatives better than other models, especially when thresholds are adjusted. Random forest has a significant advantage over XGBoost and SVM when it comes to sensitivity, which makes it particularly suitable for prediabetes detection, where minimizing missed cases is crucial. While KNN is the most effective at identifying normal cases, it lacks the discriminative power necessary to accurately classify prediabetes, illustrating that it may be more fit as a baseline or for smaller datasets.

### ROC Curves

#### Overview

**Figure 5.**
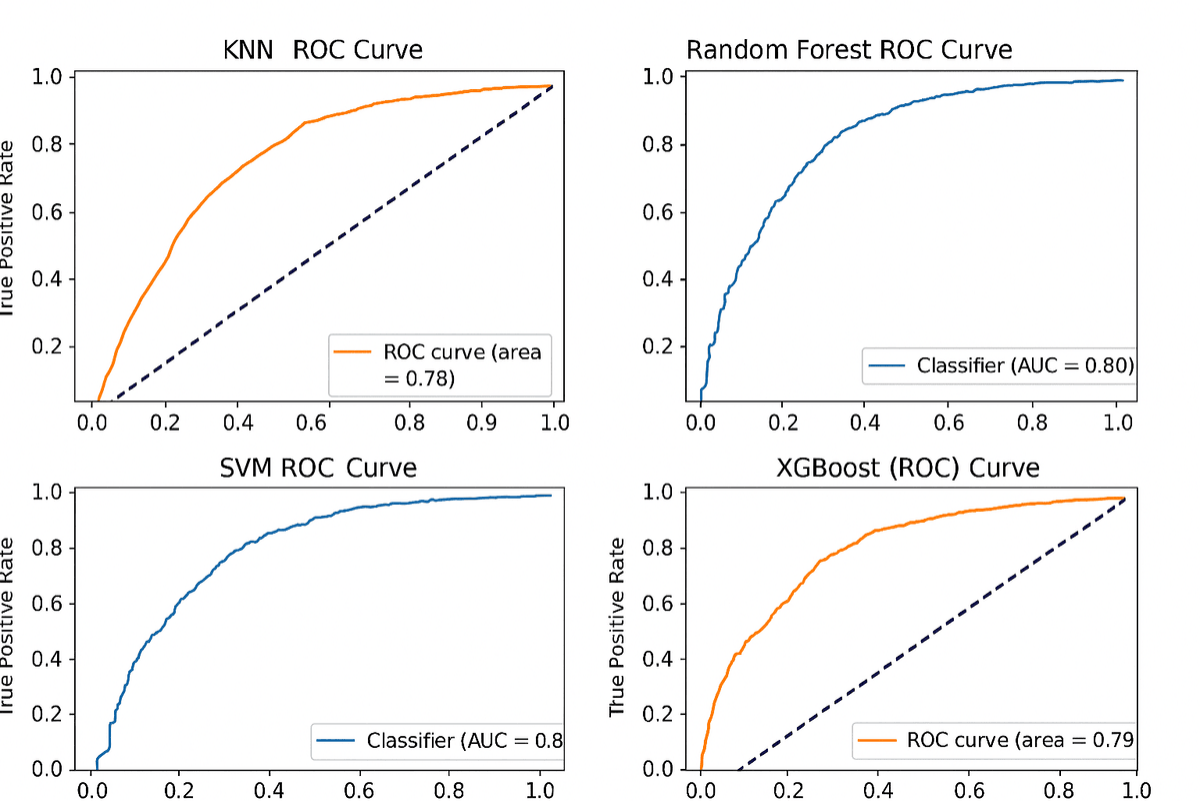
ROC curve comparison across models. KNN: *k*-nearest neighbor; SVM: support vector machine; XGBoost: extreme gradient boosting.

Figure 5 shows the ROC (receiver operating characteristic) curves for every model, further clarifies the trade-offs between sensitivity and specificity, and shows the performance of each model in terms of how well it separates between normal and prediabetic cases. The random forest model showed the most convenient ROC curve, while XGBoost and SVM also displayed powerful curves, suggesting effective categorization performance.

#### XGBoost

This classifier showed an AUC (area under the curve) of 0.79. The XGBoost ROC curve reflects a relatively good trade-off between the true positive rate (sensitivity) and false positive rate (1 – specificity), indicating that it is an effective classification model, but has some room for improvement in distinguishing classes.

#### About SVM

The SVM classifier produced a slightly lower AUC of 0.78. However, the SVM struggles slightly more with false positives, as indicated by its ROC curve, which does not consistently approach the top-left corner. Despite this, it performs reasonably well when it comes to classification.

#### Random Forest

Across the 4 models tested, the random forest model achieved the elevated AUC at 0.80. With a more pronounced upward curve, its ROC curve reflects better differentiation between positive and negative classes, showcasing outstanding classification abilities.

#### About KNNs

The KNN classifier achieved a score of 0.78, suggesting a fair rank of accuracy in the diagnosis of positive and negative cases. According to the ROC curve for the KNN model, there is a moderate trade-off between the true positive rate (sensitivity) and the false positive rate (1 – specificity). As well, there is some evidence to suggest that the KNN model has some ability to separate the 2 classes, but its shape suggests that it has room for improvement, as it does not consistently approach the top-left corner, which would indicate an ideal performance.

In a nutshell, all 4 models exhibit durable performance, with AUC values ranging from 0.78 to 0.80. The random forest model manifests as the best-performing classifier, followed closely by XGBoost, SVM, and KNN.

## Discussion

### Principal Findings

Through systematically integrating model comparison, advanced hyperparameter tuning, and interpretable feature selection techniques, we present a robust, interpretable framework for early prediabetes prediction. By combining SHAP analysis and LASSO regression, this research provides both high performance and transparency, compared to previous studies that focused solely on accuracy.

### Comparative Strengths and Limitations of Each Model

#### Overview

For prediabetes prediction, XGBoost, random forest, SVM, and KNN each show distinct strengths and weaknesses.

#### Random Forest

In terms of overall discriminative ability, the random forest model accomplished a superior cross-validated ROC-AUC score (0.9117). According to this result, random forest is a robust choice for early detection scenarios as it can generalize to different datasets well. Due to its ability to prioritize recall through threshold adjustments, 22 false negatives were reduced, but false positives increased (230). In view of this trade-off, random forest may be highly powerful when the cost of missing a prediabetic case outweighs the risk of overdiagnosis.

#### XGBoost

In evaluation, the XGBoost classifier showcased robust performance, as it attained a high precision score of 0.8128 and a balanced recall score. According to these metrics, it seems that XGBoost is particularly adept at minimizing false positives and false negatives, which is highly critical in clinical settings where diagnostic accuracy directly influences patient outcomes. The ROC-AUC score of XGBoost did not surpass that of random forest, despite its ability to balance sensitivity and specificity, making it a viable choice for routine clinical applications.

#### About SVM

With an AUC of 0.78, the SVM model ranked behind both XGBoost and random forest. Despite their superior performance in high-dimensional spaces and in datasets with clear class separation, SVM models have limited linear separability in the prediabetes dataset, impacting their discriminative power. The model has a good ROC-AUC and *F*_1_-score, with reasonable precision and recall, but when it comes to complex relationships, it lags behind the others. Optimizing feature engineering may upgrade its performance by searching alternative SVM kernels, combining nonlinear interactions, or incorporating alternative kernels.

#### About KNNs

It performed rationally well in terms of classification performance but ranked lowest in terms of accuracy among the evaluated models, with an accuracy of 70.8% and ROC-AUC of 0.78. Because of its simplicity and reliance on distance metrics, KNN is expected to have lower discriminative power than more complex models such as random forest and XGBoost. This model may be valuable as a baseline model or may be convenient for small datasets with a focus on computational efficiency. The reasonable performance of KNN is a result of its sensitivity to distance metrics and the number of neighbors (*k*), which may prevent it from catching subtle differences in detecting normal and prediabetic cases. Thus, while KNN may be beneficial in straightforward scenarios, it does not have the same level of precision and recall as more sophisticated models.

### Impact of Feature Selection

Feature selection played a crucial role in optimizing the models’ performance by focusing on the main relevant predictors. LASSO regression was used to characterize the prime features across models, with BMI, age, LDL-C, and HDL-C consistently emerging as important risk factors for prediabetes. In addition to improving the interpretability of the models, this approach also improved the predictive accuracy by reducing overfitting. The strict feature selection process warranted that the models stayed efficient while maintaining high classification power.

### Confusion Matrix and Threshold Analysis

The performance metrics were significantly influenced by adjusting decision thresholds, especially for random forest and XGBoost. A threshold adjustment in random forest minimized the risk of missed diagnoses by reducing false negatives (22 cases). Even so, this came at the expense of a boosted number of false positives (230 cases), suggesting a trade-off between recall and precision. XGBoost, while less sensitive to threshold changes, maintained a balanced approach, limiting both false positives and false negatives effectively. As a result of these outcomes, threshold tuning plays an important role in optimizing model performance for specific clinical applications, such as prioritizing recall in high-risk populations to avoid disease progression.

### Clinical Implications

The results suggest that XGBoost and random forest are the most promising models for enhancing prediabetes diagnosis, given their ability to generalize across different datasets and include reliable classification performance. The higher ROC-AUC score achieved over random forest (91.17%) reflects its potential for widespread use in clinical settings, especially where minimizing the risk of missed cases is crucial. The powerful performance of XGBoost among diverse metrics also highlights its practicality for routine screening, where both false positives and false negatives need to be minimized. By adjusting model thresholds, clinicians can customize diagnostic strategies to meet individual patient needs, such as increasing sensitivity for at-risk patients. Even though SVMs and KNNs do not outperform the best models, they still provide useful insights, especially when data dimensionality or simplicity are important factors.

### Conclusions

ML models, specifically random forest and XGBoost, have been found to be most sensitive to prediabetes risk assessment, and their performance has powerful discriminative power and high ROC-AUC scores. Combined with feature selection techniques such as LASSO regression, these models offer worthy insights into essential prediabetes predictors, such as BMI, age, and HDL-C. Based on the ROC and AUC analyses, all models—XGBoost, SVM, random forest, and KNN—are viable options for predicting prediabetes. Random forests are robust classifiers because of their ensemble nature, which reduces overfitting and enhances generalizability. SVM and XGBoost also produce competitive results, suggesting their classification abilities can be improved with further parameter tuning. With systematic exploratory data analysis and feature selection, these models can become reliable tools for detecting early prediabetes and offering pathways for optimizing them.

To confirm the generalizability of these models, future research should include validating them in diverse populations, adding biomarkers and genetics to improve prediction accuracy, and integrating these models into clinical decision support systems to assess risk in real time. These models contribute to more accurate and timely diagnosis of prediabetes, promoting timely intervention and ultimately improving health outcomes.

## Abbreviations

AUC: area under the curve
HDL-C: high-density lipoprotein cholesterol
KNN: *k*-nearest neighbor
LASSO: Least Absolute Shrinkage and Selection Operator
LDL-C: low-density lipoprotein cholesterol
ML: machine learning
PCA: principal component analysis
ROC: receiver operating characteristic
ROC-AUC: receiver operating characteristic area under the curve
SHAP: Shapley Additive Explanations
SMOTE: Synthetic Minority Oversampling Technique
SVM: support vector machine
XGBoost: extreme gradient boosting

## References

[1] Liu Y, Feng W, Lou J (2023). Performance of a prediabetes risk prediction model: a systematic review. Heliyon.

[2] Schwartz JL, Tseng E, Maruthur NM, Rouhizadeh M (2022). Identification of prediabetes discussions in unstructured clinical documentation: validation of a natural language processing algorithm. JMIR Med Inform.

[3] Hathaway QA, Roth SM, Pinti MV (2019). Machine-learning to stratify diabetic patients using novel cardiac biomarkers and integrative genomics. Cardiovasc Diabetol.

[4] De Silva K, Jönsson D, Demmer RT (2020). A combined strategy of feature selection and machine learning to identify predictors of prediabetes. J Am Med Inform Assoc.

[5] Dwyer DB, Falkai P, Koutsouleris N (2018). Machine learning approaches for clinical psychology and psychiatry. Annu Rev Clin Psychol.

[6] Talari P, N B, Kaur G (2024). Hybrid feature selection and classification technique for early prediction and severity of diabetes type 2. PLoS ONE.

[7] Liu Q, Zhou Q, He Y, Zou J, Guo Y, Yan Y (2022). Predicting the 2-year risk of progression from prediabetes to diabetes using machine learning among Chinese elderly adults. J Pers Med.

[8] Abbas M, Mall R, Errafii K (2021). Simple risk score to screen for prediabetes: a cross-sectional study from the Qatar Biobank cohort. J Diabetes Investig.

[9] Hu Y, Han Y, Liu Y (2023). A nomogram model for predicting 5-year risk of prediabetes in Chinese adults. Sci Rep.

[10] Yu LP, Dong F, Li YZ (2022). Development and validation of a risk assessment model for prediabetes in China national diabetes survey. World J Clin Cases.

[11] Dong W, Tse TYE, Mak LI (2022). Non-laboratory-based risk assessment model for case detection of diabetes mellitus and pre-diabetes in primary care. J Diabetes Investig.

[12] Liaw LCM, Tan SC, Goh PY, Lim CP (2025). A histogram SMOTE-based sampling algorithm with incremental learning for imbalanced data classification. Inf Sci.

[13] Raju VNG, Lakshmi KP, Jain VM, Kalidindi A, Padma V Study the influence of normalization/transformation process on the accuracy of supervised classification.

[14] Da Poian V, Theiling B, Clough L (2023). Exploratory data analysis (EDA) machine learning approaches for ocean world analog mass spectrometry. Front Astron Space Sci.

[15] Saxena R, Sharma SK, Gupta M, Sampada GC (2022). A novel approach for feature selection and classification of diabetes mellitus: machine learning methods. Comput Intell Neurosci.

[16] Noaro G, Cappon G, Vettoretti M, Sparacino G, Favero SD, Facchinetti A (2021). Machine-learning based model to improve insulin bolus calculation in type 1 diabetes therapy. IEEE Trans Biomed Eng.

[17] Gollapalli M, Alansari A, Alkhorasani H (2022). A novel stacking ensemble for detecting three types of diabetes mellitus using a Saudi Arabian dataset: pre-diabetes, T1DM, and T2DM. Comput Biol Med.

[18] Jia W, Sun M, Lian J, Hou S (2022). Feature dimensionality reduction: a review. Complex Intell Syst.

[19] Hasan MK, Alam MA, Das D, Hossain E, Hasan M (2020). Diabetes prediction using ensembling of different machine learning classifiers. IEEE Access.

[20] Liu CH, Chang CF, Chen IC (2024). Machine learning prediction of prediabetes in a young male Chinese cohort with 5.8-year follow-up. Diagnostics (Basel).

[21] Alzyoud M, Alazaidah R, Aljaidi M (2024). Diagnosing diabetes mellitus using machine learning techniques. Int J Data Netw Sci.

[22] Olisah CC, Smith L, Smith M (2022). Diabetes mellitus prediction and diagnosis from a data preprocessing and machine learning perspective. Comput Methods Programs Biomed.

[23] Diranisha V, Triayudi A, Komalasari RT (2024). Implementation of k-nearest neighbour (KNN) algorithm and random forest algorithm in identifying diabetes. SAGA J Technol Inform Syst.

[24] Yennimar Y, Rasid A, Kenedy S (2023). Implementation of support vector machine algorithm with hyper-tuning randomized search in stroke prediction. J Sist Inf Ilmu Komput Prima.

[25] Yates LA, Aandahl Z, Richards SA, Brook BW (2023). Cross validation for model selection: a review with examples from ecology. Ecol Monogr.

